# Supplementary material for: Test–retest reliability and minimal detectable change of pulse wave velocity and flow‐mediated dilation in paretic and non‐paretic limbs of older adults post‐stroke
Source: Physiol Rep. 2026 Oct 19;14(20):e71138. doi: 10.14814/phy2.71138 (PMC13680993; doi:10.14814/phy2.71138)
Supplement: Supplementary file 1 [file PHY2-14-e71138-s001.docx]

| Appendix A. Relative and absolute reliability of relative FMD by paretic and non-paretic limbs. | | | | | | | | | |
| --- | --- | --- | --- | --- | --- | --- | --- | --- | --- |
| **Measures** | **N** | **Test 1 (SD)** | **Test 2 (SD)** | **SD_Pooled_** | **ICC_2,1_ (95% CI)** | **SEM** | **MDC_68%_** | **MDC_90%_** | **MDC_95%_** |
| Paretic | 10 | 5.88 (3.58) | 4.53 (4.34) | 3.98 | 0.78 (0.35, 0.94) | 1.86 | 2.63 | 4.32 | 5.15 |
| Non-Paretic | 10 | 4.21 (4.32) | 5.93 (5.63) | 5.02 | 0.70 (0.22, 0.92) | 2.74 | 3.87 | 6.36 | 7.58 |
| Abbreviations. FMD: flow mediated dilation; ICC_2,1_: intra-class correlation coefficient; MDC: minimal detectable change; SEM: standard error of measurement. | | | | | | | | | |
